# Factors associated with acute myocardial infarction in older patients after hospitalization with community-acquired pneumonia: a cross-sectional study

**DOI:** 10.1186/s12877-021-02056-6

**Published:** 2021-02-09

**Authors:** Yu Kang, Xiang-Yang Fang, Dong Wang, Xiao-Juan Wang

**Affiliations:** 1 Department of Geriatric Medicine, Beijing Chao-Yang Hospital, Capital Medical University, 8 Gongren Tiyuchang Nanlu, Chaoyang District, Beijing, 100020 China; 2 Department of Respiratory and Critical Care Medicine, Beijing Chao-Yang Hospital, Capital Medical University, Beijing, China

**Keywords:** Community-acquired pneumonia, Pneumonia, Acute myocardial infarction, Myocardial infarction, Geriatric

## Abstract

**Background:**

Community-acquired pneumonia (CAP) and acute myocardial infarction cardiovascular (AMI) are two important health issues in older patients. Little is known regarding characteristics of AMI in older patients hospitalized for CAP. Therefore, we investigated the prevalence, characteristics compared with younger patients, impact on clinical outcomes and risk factors of AMI during hospitalization for CAP in geriatric patients.

**Methods:**

Eleven thousand nine adult inpatients consisted of 5111 patients≥65 years and 5898 patients< 65 years in respiratory ward diagnosed with CAP were retrospectively analyzed by electronic medical records.

**Results:**

159 (3.1%) older patients in respiratory ward experienced AMI during hospitalization for CAP. AMI were more frequently seen in patients≥65 years compared with patients< 65 years (3.1% vs. 1.0%). Patients≥65 years who experienced AMI during hospitalization for CAP had higher percentage of respiratory failure (*P* = 0.001), hypertension (*P* = 0.008), dyspnea (*P* = 0.046), blood urea nitrogen (BUN) ≥7 mmol/L (*P* < 0.001), serum sodium< 130 mmol/L (*P* = 0.005) and had higher in-hospital mortality compared to patients< 65 years (10.1% vs. 6.6%). AMI was associated with increased in-hospital mortality (odds ratio, OR, with 95% confidence interval: 1.49 [1.24–1.82]; *P* < 0.01). Respiratory failure (OR, 1.34 [1.15–1.54]; *P* < 0.01), preexisting coronary artery disease (OR, 1.31[1.07–1.59]; *P* = 0.02), diabetes (OR, 1.26 [1.11–1.42]; P = 0.02) and BUN (OR, 1.23 [1.01–1.49]; *P* = 0.04) were correlated with the occurrence of AMI in the older patients after hospitalization with CAP.

**Conclusions:**

The incidence of AMI during CAP hospitalization in geriatric patients is notable and have an impact on in-hospital mortality. Respiratory failure, preexisting coronary artery disease, diabetes and BUN was associated with the occurrence of AMI in the older patients after hospitalization with CAP. Particular attention should be paid to older patients with risk factors for AMI.

## Background

The growing proportion of older adults provides a compelling reason for an increased focus on public health problems of older people. Community-acquired pneumonia (CAP) and acute myocardial infarction cardiovascular (AMI) are two major public health issues in older patients. It is estimated that incidence of CAP patients≥65 years old was 140 cases per 10,000 persons per year and 105 cases per 10,000 for hospitalized [1]. The mortality rate for CAP has been decreasing after the introduction of antibiotics, even so, high mortality still in the older patients. Mortality in geriatric patients with CAP may be 25% higher than in the general population [2, 3]. The incidence and mortality risk in CAP are linked to increasing age and the presence of age-related comorbidities and complications. Thus, there is a clear need to recognize the life-threatening complications among geriatric patients with CAP.

A link between acute infections and the development of cardiovascular complications has been proposed [4–9]. Pneumonia contributes to the acute worsening of pre-existing cardiac conditions and can trigger new cardiac events. Moreover, a higher likelihood of a poorer outcome in a patient with CAP complicated by an acute cardiovascular event [10]. Cardiovascular complications represent a heavy burden on the course and outcomes of patients admitted to hospital with CAP. Recent clinical observations suggest that acute cardiovascular complications are more frequent in high-risk CAP patients. The older adults are at high risk of CAP. An increase in pneumonia hospital admissions and potentially deaths mainly due to the ageing population [11], a population that is also at the highest risk for cardiovascular diseases. Cardiovascular complications after pneumonia including new or worsening heart failure, new or worsening arrhythmias and AMI. Hitherto, reports of cardiovascular complications after CAP were mostly focus on heart failure, few about AMI, which is a life-threatening complication. In particularly, systematically studied of geriatric patients are unavailable to date.

Despite AMI after CAP in geriatric patients is a worthy concern, little is known regarding the clinical characteristics of the older adults. Therefore, our study aimed to determine the prevalence, characteristics compared with younger patients, impact on clinical outcomes and risk factors of AMI during hospitalization for CAP in geriatric patients.

## Methods

### Study population and data collection

We identified a total of the 11,009 patients who were diagnosed with CAP and age ≥ 18 years was hospitalized in the respiratory ward of Beijing Chao-yang Hospital between June 01, 2012 and June 30, 2020, Of these, there were 5111 patients≥65 years. Beijing Chao-Yang Hospital has 1900 beds including 196 beds in the respiratory department. Beijing Chao-Yang Hospital not only has the Beijing Institute of respiratory diseases, well-recognized at the national level, but also houses one of the key construction geriatrics wards in Beijing.

The clinical information data from all patients were extracted from the electronic medical records. The study protocol was approved by the Institutional Review Board for Human Studies of Beijing Chaoyang Hospital, Beijing, China. The following variables were collected: age, sex, smoking, co-morbidity, clinical symptoms, clinical condition (body temperature, respiratory rate, blood pressure, heart rate, mental status and percutaneous oxygen saturation) and laboratory findings on hospital admission. In our center, clinical information collection and laboratory examinations were performed during the first 24 h after admission and according to standards of practice.

### Diagnosis and definitions

#### Diagnosis of CAP

CAP was diagnosed in our center according with the IDSA/ATS (Infectious Diseases Society of America and the American Thoracic Society) guidelines [12]: At least one of the clinical symptoms: cough, sputum, fever, dyspnea, and pleuritic chest pain; at least more than one finding of coarse crackles by auscultation or inflammatory biomarkers elevated; a new infiltrate be found on chest radiograph.

#### Diagnosis of AMI

AMI that occurred at any time during hospitalization for pneumonia was included. In our center, AMI were diagnosed according to the following criteria: detection of troponin with at least one value above the 99th percentile of the upper reference limit together with evidence of myocardial ischaemia with at least one of the following: (1) symptoms of ischaemia: including chest pain, chest tightness and other symptoms; (2) acute electrocardiographic changes of new ischaemia (ST segment and T wave changes or new left bundle branch block); (3) new pathological Q-waves in the electrocardiographic; or (4) imaging evidence of new loss of viable myocardium or new regional wall motion abnormality [13]. Diagnosis of AMI was done by cardiologist consultation in the respiratory ward.

### Other clinical characteristics

The main outcome was the in-hospital mortality. In-hospital mortality was depended on vital status at discharge including death by any cause occurred during hospital stay. In addition, we also investigated the length of hospital stay, length of hospital stay was considered as the number of days from the date of admission to the date of discharge. The proportion of patients who developed respiratory failure or required ventilator use in patients with and without AMI during hospitalization were compared. Diagnostic criteria for respiratory failure were as follows: PO_2_ (pressure of oxygen) < 60 mmHg, with or without PCO_2_ (pressure of carbon dioxide) ≥45 mmHg, under room air according to blood gas analysis on admission. Ventilator use including invasive mechanical ventilation (IMV) or non-invasive ventilation (NIV).

### Statistical analysis

Categorical variables were described using counts and percentages, and groups were compared using a Chi-square test or Fisher’s exact probability test. Continuous variables were presented as means and standard deviations, and significant differences between two groups were determined with a Student’s *t-*test. For non-normally distributed data, median and interquartile ranges were used to describe the features, while comparisons of the two sets were performed using a Mann-Whitney U test. To determine the factors associated with the occurrence of AMI during hospitalization for pneumonia in the older patients and investigated the relationship between AMI and the in-hospital mortality, logistic regression analysis was performed. The odds ratios (OR) with 95% confidence intervals (CI) were presented.

The statistical analyses of data were performed by using SPSS 20.0 (SPSS Inc., Chicago, IL, USA) and R software (version 3.3.2) with the corresponding R packages. All tests were two-sided, and a value of *P* < 0.05 was considered statistically significant.

## Results

### Clinical characteristics of geriatric patients who experiencing AMI and those who without AMI during CAP hospitalization

There were 5111 patients in the respiratory ward hospitalized for CAP and aged≥65 years, 159 experienced AMI during hospitalization. The incidence of AMI in geriatric patients during CAP hospitalization in our study was 3.1%.

The clinical characteristics of geriatric patients who experiencing AMI and those who without AMI during CAP hospitalization are shown in Table 1. Comparison to patients without AMI, those geriatric patients with AMI during CAP hospitalization were older (*P* < 0.001) and showed a higher prevalence of respiratory failure (*P* = 0.001) and required ventilator use (*P* < 0.001), had longer hospital stays (*P* = 0.023). Additionally, a high proportion of patients presenting chief complaint of chest pain or dyspnea (*P* = 0.030, *P* < 0.001, respectively), abnormal blood pressure (*P* < 0.001), body temperature (*P* = 0.003), heart rate ≥ 125 bpm (*P* < 0.001), blood urea nitrogen (BUN) ≥7 mmol/L (*P* < 0.001), blood platelet (PLT) < 10.0 × 10^9^/L (*P* < 0.001), serum sodium< 130 mmol/L (*P* < 0.001) or blood glucose≥14 mmol/L (*P* < 0.001) on hospital admission was higher in patients who experiencing AMI compared with those who without AMI during hospitalized for CAP. Moreover, there were a higher percentage of males (*P* = 0.001), preexisting coronary artery disease (*P* < 0.001), hypertension (*P* = 0.001), hypercholesterolemia (*P* < 0.001), diabetes (*P* < 0.001), history of chronic heart failure (*P* = 0.003) or cerebrovascular disease (*P* = 0.001) in patients experiencing AMI during CAP hospitalization.

**Table 1:** Characteristics of geriatric patients experiencing AMI and patients without AMI

Characteristic	Patients experiencing AMI	Patients Without AMI	***P*** value^a^
	*n* = 159	*n* = 4952	
**Age**, years	76.7 ± 6.9	73.9 ± 6.6	< 0.001
**Male**, n (%)	112 (62.1)	2847 (57.5)	0.001
**Comorbid conditions,** n (%)			
Smoking	67 (42.1)	2618 (52.9)	0.012
Preexisting coronary artery disease	67 (42.1)	877 (17.7)	< 0.001
COPD	26 (16.4)	667 (13.5)	0.906
Lung cancer	16 (10.1)	463 (9.3)	0.782
Diabetes	65 (40.9)	1031 (20.8)	< 0.001
Hypertension	99 (62.3)	2409 (48.6)	0.001
Hypercholesterolemia	29 (18.2)	432 (8.7)	< 0.001
Chronic heart failure	6 (3.8)	40 (0.8)	0.003
Cerebrovascular disease	22 (13.8)	327 (6.6)	0.001
Chronic renal failure	6 (3.8)	85 (1.7)	0.063
Chronic liver disease	6 (3.8)	145 (2.9)	0.474
**Clinical symptoms of chief complaint on admission**, n (%)			
Fever	95 (59.7)	3170 (64.0)	0.276
Cough and expectoration	79 (49.7)	2362 (47.7)	0.629
Chest pain	13 (8.2)	222 (4.5)	0.030
Dyspnea	42 (26.4)	693 (13.9)	< 0.001
Duration of symptoms	6.9 ± 6.7	7.17 ± 7.0	0.927
**Clinical data**, n (%)			
NIV/IMV	17 (10.7)	155 (3.1)	< 0.001
Respiratory failure	41 (25.8)	776 (15.7)	0.001
Impaired consciousness	3 (1.9)	40 (0.8)	0.150
Respiratory rate ≥ 30/min	4 (2.5)	55 (1.1)	0.110
SBP < 90 mmHg or DBP ≤ 60 mmHg	19 (11.9)	12 (0.2)	< 0.001
T < 36 °C or ≥ 40 °C	2 (1.3)	4 (0.1)	0.003
Heart rate ≥ 125 bpm.	2 (1.3)	45 (0.9)	< 0.001
BUN ≥7 mmol/L	95 (59.7)	1412 (28.5)	< 0.001
WBC < 4.0 × 10^9^/L or ≥ 10.0 × 10^9^/L	71 (44.7)	2319 (46.8)	0.323
PLT < 10.0 × 10^9^/L	14 (8.8)	7 (0.1)	< 0.001
PH < 7.35	15 (9.4)	447 (9.0)	0.471
Serum sodium < 130 mmol/L	50 (31.4)	903 (18.2)	< 0.001
HCT < 30%	21 (13.2)	491 (9.9)	0.112
Blood glucose ≥14 mmol/L	11 (6.9)	74 (1.5)	< 0.001
Pleural effusion	122 (76.7)	4089 (82.6)	0.071
**Pathogens**			
Bacterial pneumonia	126 (79.2)	4011 (81.0)	0.952
Viral pneumonia	3 (1.9)	64 (1.3)	0.465
Fungal pneumonia	8 (5.0)	140 (2.8)	0.140
**Death in hospital**, n (%)	16 (10.1)	118 (2.4)	< 0.001
**Length of stay,** days	13.8 ± 8.9	12.1 ± 9.8	0.023

*CAP* Community - acquired pneumonia, *COPD* Chronic obstructive pulmonary disease, *IMV* Invasive mechanical ventilation, *NIV* Non - invasive ventilation

*SBP* Systolic blood pressure, *DBP* Diastolic blood pressure, *T* Body temperature, *Bpm* Beats per minute, *BUN* Blood urea nitrogen, *WBC* White blood cell

*PLT* Blood platelet, *PH* Potential of hydrogen. *HCT* Hematocrit

^a^For comparisons between AMI group and Without AMI group. Data are presented as mean (standard deviation) or %. AMI: acute myocardial infarction

### Comparison of the characteristics between older patients and non-older patients who experiencing AMI during CAP hospitalization

**Fig. 1:**
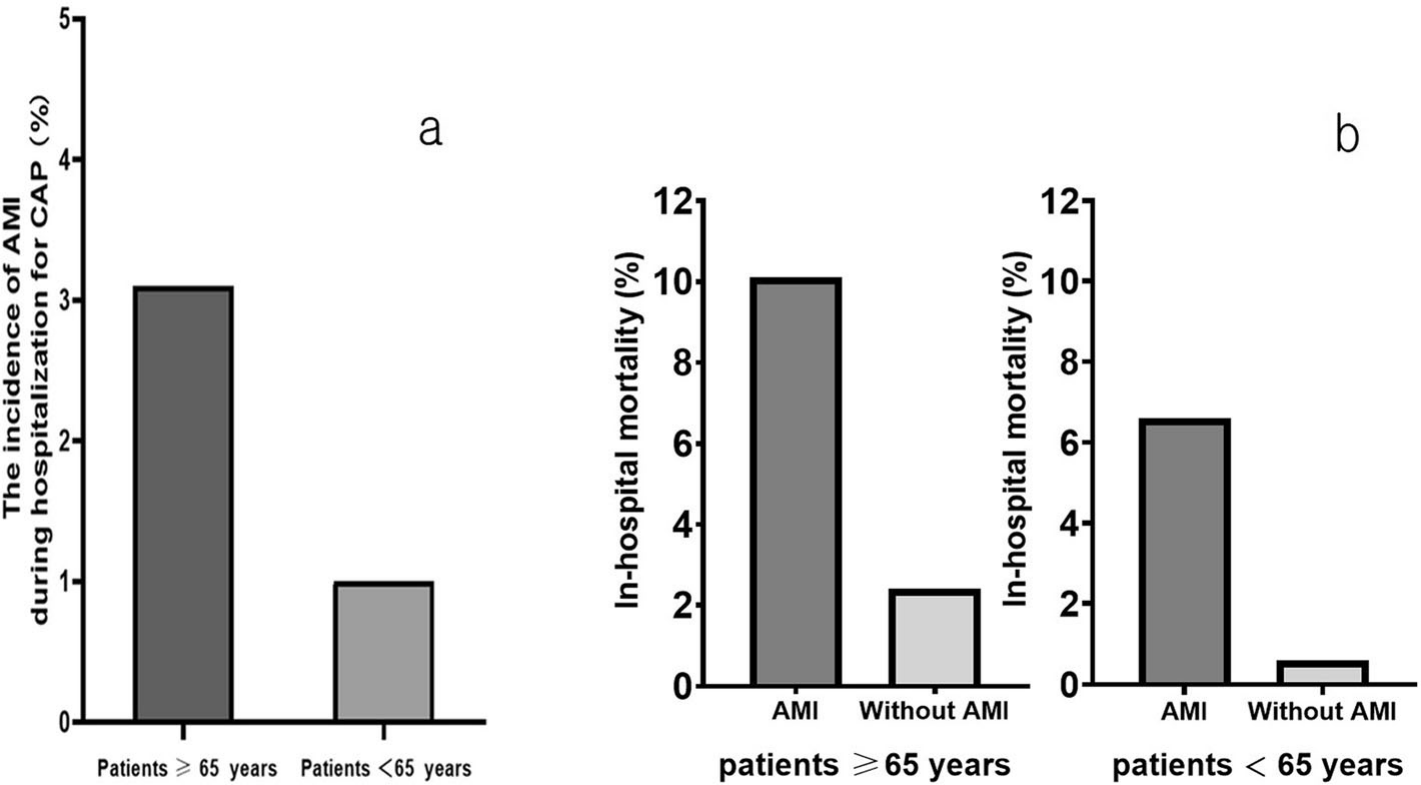
Comparison of the characteristics between elderly patients and non-elderly patients. **a** The incidence of AMI during hospitalization for CAP and **b** The in-hospital mortality. AMI: myocardial infarction cardiovascular; CAP: community-acquired pneumonia

We identified a total of the 11,009 patients who were diagnosed with CAP and age ≥ 18 years was hospitalized in the respiratory ward, consisted of 5111 older patients≥65 years and 5898 patients< 65 years. One hundred fifty-nine patients≥65 years and 61 patients< 65 years experienced AMI during hospitalization for CAP in respiratory ward. As shown in Fig. 1, AMI after CAP were more frequently seen in older patients (3.1% vs. 1.0%). In a further analysis, patients≥65 years were compared with those patients< 65 years experiencing AMI during CAP hospitalization in the respiratory ward (Table 2). We noted that higher proportion patients suffer from respiratory failure in older patients (*P* = 0.001). More patients≥65 years had hypertension (*P* = 0.008), dyspnea (*P* = 0.046), BUN≥7 mmol/L (*P* < 0.001) or serum sodium< 130 mmol/L (*P* = 0.005). On the other hand, those patients< 65 years had higher percentage of male (*P* = 0.012), smoking history (*P* < 0.001), diabetes (*P* < 0.001), hypercholesterolemia (*P* = 0.002), chest pain (*P* = 0.019) or abnormal white blood cell count (*P* = 0.002). As shown in Fig. 1, patients≥65 years had a higher in-hospital mortality compared to patients< 65 years (10.1% vs. 6.6%).

**Table 2:** Comparison the characteristics between ≥65 years and < 65 years patients experiencing AMI

Characteristic	patients ≥ 65 years *n* = 159	patients < 65 years *n* = 61	***P*** value^a^
**Age**, years	76.7 ± 6.9	57.8 ± 5.2	< 0.001
**Male**, n (%)	112 (62.1)	56 (91.8)	0.012
**Comorbid conditions**, n (%)			
Smoking	67 (42.1)	54 (88.5)	< 0.001
Preexisting coronary artery disease	67 (42.1)	39 (63.9)	0.768
COPD	26 (16.4)	9 (14.8)	0.968
Lung cancer	16 (10.1)	3 (4.9)	0.203
Diabetes	65 (40.9)	43 (70.5)	< 0.001
Hypertension	99 (62.3)	29 (47.5)	0.008
Hypercholesterolemia	29 (18.2)	24 (39.3)	0.002
Chronic heart failure	6 (3.8)	1 (1.6)	0.376
Cerebrovascular disease	22 (13.8)	4 (6.6)	0.115
Chronic renal failure	6 (3.8)	1 (1.6)	0.376
Chronic liver disease	6 (3.8)	5 (8.2)	0.300
**Clinical symptoms of chief complaint on admission**, n (%)			
Fever	95 (59.7)	46 (75.4)	0.224
Cough and expectoration	79 (49.7)	40 (65.6)	0.143
Chest pain	13 (8.2)	12 (42.1)	*0.018*
Dyspnea	42 (26.4)	9 (14.8)	0.046
Duration of symptoms	6.9 ± 6.7	5.2 ± 3.8	0.589
**Clinical data**, n (%)			
NIV/IMV	17 (10.7)	4 (6.6)	0.255
respiratory failure	41 (25.8)	6 (9.8)	0.001
Impaired consciousness	3 (1.9)	0 (0.0)	0.376
Respiratory rate ≥ 30/min	4 (2.5)	1 (1.6)	0.575
SBP < 90 mmHg or DBP ≤60 mmHg	19 (11.9)	7 (11.5)	0.596
T < 36 °C or ≥ 40 °C	2 (1.3)	2 (3.3)	0.308
Heart rate ≥ 125 bpm.	2 (1.3)	1 (1.6)	0.108
BUN ≥7 mmol/L	95 (59.7)	14 (22.9)	0.000
WBC < 4.0 × 109/L or ≥ 10.0 × 109/L	71 (44.7)	41 (67.2)	0.002
PLT < 10.0 × 109/L	14 (8.8)	5 (8.2)	0.562
PH < 7.35	15 (9.4)	2 (3.3)	0.106
Serum sodium < 130 mmol/L	50 (31.4)	8 (13.1)	0.005
HCT < 30%	21 (13.2)	6 (9.8)	0.333
Blood glucose ≥14 mmol/L	11 (6.9)	6 (9.8)	0.319
Pleural effusion	122 (76.7)	53 (86.9)	0.482
**Pathogens**			
Bacterial pneumonia	126 (79.2)	49 (80.3)	0.892
Viral pneumonia	3 (1.9)	2 (3.3)	0.425
Fungal pneumonia	8 (5.0)	3 (4.9)	0.638
**Died during hospital stay**, n (%)	16 (10.1)	4 (6.6)	0.301
**Length of stay,** days	13.8 ± 8.9	12.3 ± 7.4	0.328

*AMI* Acute myocardial infarction, *CAP* Community-acquired pneumonia, *COPD* Chronic obstructive pulmonary disease, *SBP* Systolic blood pressure, *DBP* Diastolic blood pressure, *T* Body temperature, *bpm* beats per minute, *BUN* Blood urea nitrogen, *WBC* White blood cell, *PLT* Blood platelet, *PaO2* arterial oxygen tension

*SPO2* pulse oxygen saturation, *PH* Potential of hydrogen, *IMV* Invasive mechanical ventilation, *NIV* Non-invasive ventilation

^a^For comparisons between ≥65 years group and < 65 years group. Data are presented as mean (standard deviation) or %

### Association between incidence of AMI during CAP hospitalization and in-hospital mortality

**Fig. 2:**
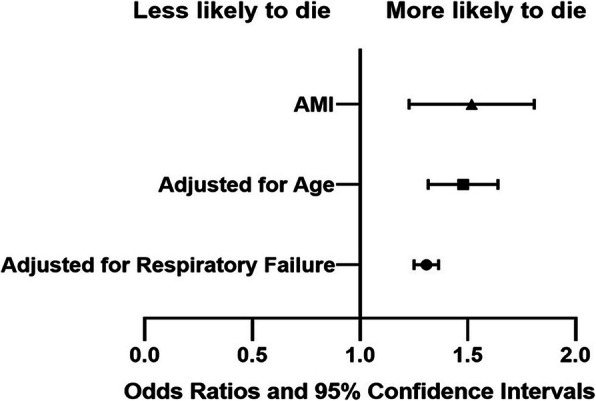
The in-hospital mortality risk in geriatric patients with CAP who developed AMI versus those who did not. CAP: community-acquired pneumonia; AMI: myocardial infarction cardiovascular

As shown in Fig. 1, in-hospital mortality was higher among older patients hospitalized with CAP in the respiratory ward who developed AMI compared to those who did not (10.1%vs.2.4%). We also investigated the association between incidence of AMI during CAP hospitalization and in-hospital mortality using logistic regression (Fig. 2), the development of AMI was associated with a increase in the risk of death during CAP hospitalization, AMI during hospitalization showed an OR for in-hospital mortality of 1.49 (95%CI: 1.24–1.82; *P* < 0.01). This association remained significant after adjustment for age (OR, 1.47; 95%CI, 1.32–1.64; P < 0.01) and after adjustment for respiratory failure (OR, 1.31; 95% CI, 1.25–1.36; P < 0.01). Conversely, we did not identify evidence of an association between in-hospital mortality and AMI in people aged < 65 years, similar meaningful statistical results could not be obtained.

### Risk factors correlated with the occurrence of AMI during hospitalization for pneumonia in the older patients

The logistic regression analysis of risk factors for AMI during hospitalization in the older patients is shown in Table 3. Input variables for logistic regression analysis were selected from significant variables obtained from the univariate analysis. In 5111 older patients≥65 years, respiratory failure (OR, 1.34; 95% CI, 1.15–1.54; *p* < 0.01), preexisting coronary artery disease (OR, 1.31; 95% CI,1.07–1.59; *p* = 0.02), diabetes (OR, 1.26; 95% CI,1.11–1.42; p = 0.02), BUN (OR, 1.23; 95% CI, 1.01–1.49; *p* = 0.04) were significantly correlated with the occurrence of AMI, as shown in Table 3.

**Table 3:** Factors for AMI during hospitalization in geriatric patients with CAP^a^

Characteristics	OR (95% CI)	***P*** value
**Respiratory failure**	1.34 (1.15–1.54)	< 0.01
**Preexisting coronary artery disease**	1.31 (1.07–1.59)	0.02
**Diabetes**	1.26 (1.11–1.42)	0.02
**BUN**	1.23 (1.01–1.49)	0.04

^a^Variables in the logistic regression that did not have a significant independent association with AMI in both model were: Age, Male, Hypertension, Hypercholesterolemia, SBP, WBC, Serum sodium and Impaired consciousness. *OR* Odds ratio, *Cl* Confidence interval. *AMI* Acute myocardial infarction, *CAP* Community-acquired pneumonia, *BUN* Blood urea nitrogen, *SBP* Systolic blood pressure, *WBC* White blood cell, *HCT* Hematocrit

## Discussion

In this study, we found that 159 (3.1%) older patients experienced AMI during hospitalization for CAP. The incidence of AMI during CAP hospitalization in older patients was 3.1% in our study. Hitherto, systematically studied of AMI during CAP hospitalization in geriatric population are unavailable to date. The incidence of acute coronary syndromes (ACS) in previous studies of adult CAP inpatients has varied widely, the frequency in the range between 0.7 and 11% [8, 9, 14–16]. One meta-analysis suggested the pooled ACS event rates after CAP was 4.5% [17]. A few studies reported only on myocardial infarction. A 3.1% incidence of myocardial infarction was mentioned in a study of cardiac complications in CAP inpatients reported by Vicente et al. [18] ﻿Perry et al. [5] reported a 1.5% 90-day incidence of myocardial infarction ﻿following hospital admission for pneumonia, which has the largest number of cases in previous studies about cardiovascular events after pneumonia with 50,119 patients. Compared with earlier published data, we demonstrate that AMI occurred during hospitalization in a limited but significant proportion of older adults hospitalized due to CAP.

This study is the first to investigate the characteristics of older patients who experiencing AMI during CAP hospitalization. We faces the day-to-day challenges of aging. Hence, in a further analysis, patients≥65 years were compared with those patients< 65 years experiencing AMI during CAP hospitalization in our study. AMI is more prevalent in geriatric patients with CAP than in the general population, the incidence of AMI during hospitalization in older patients (3.1%) was nearly almost triple versus non-older patients (1.0%) in our study. We noted that higher proportion patients suffer from respiratory failure in older patients. Symptoms in the older patients were more of a dyspnea, unlike chest pain in the younger patients. Usually, chest pain is considered as a typical symptom of AMI, however, symptoms in the older patients were not always typical. Since the clinical presentation of AMI in the older patients may be atypical, clinicians should suspect AMI in older patients presenting symptoms such as dyspnea. The older patients experiencing AMI had more history of hypertension, and younger patients had more diabetes and hyperlipidemia. In laboratory findings, BUN≥7 mmol/L and serum sodium < 130 mmol/L were more frequently present in the older patients. Identify the characteristics of the older patients is beneficial to clinical diagnosis, evaluation and individual-based treatment.

In-hospital mortality was significantly higher among those who experienced AMI in comparison patients who did not during hospitalization in older patients (10.1% vs. 2.4%). Moreover, in patients who experiencing AMI during CAP hospitalization, patients≥65 years had a higher in-hospital mortality compared to patients< 65 years (10.1% vs. 6.6%). AMI associated with increased in-hospital mortality of geriatric patients with CAP. The development of AMI was associated with a nearly 50% increase in the risk of death during hospitalization (OR = 1.49; 95% CI: 1.24–1.82; *P* < 0.001). This association remained significant even after adjustment for age and for respiratory failure. Conversely, we did not identify evidence of an association between in-hospital mortality and AMI in people aged < 65 years, that could be due to the incidence of AMI and the number of deaths in the non-older patients were low. Recognize the life-threatening complications among geriatric patients with CAP conducive to clinical decision-making process.

Another important aspect of our study was the identification of risk factors associated with the occurrence of AMI during CAP hospitalization in geriatric patients. Risk factors of ACS after CAP were reported only in three previous studies, possible risk factors included older age, congestive heart failure or previous myocardial infarction, female sex, severe sepsis, chronic obstructive pulmonary disease, chronic kidney or liver disease [9, 19, 20]. Through logistic regression analysis in our study, we demonstrated that preexisting coronary artery disease, diabetes, respiratory failure and BUN were significantly correlated with the occurrence of AMI. It is interesting to note that the both traditional and non-traditional cardiovascular risk factors were probably associated with the occurrence of AMI. An increased serum BUN levels suggested acute kidney injury, which is common during pneumonia. Renal insufficiency and diabetes are acknowledged risk factors for myocardial infarction [21, 22]. BUN is one indicator of the severity of pneumonia. More important however, is our finding that respiratory failure was an important factor associated with the occurrence of AMI. Respiratory failure indicates more severe disease status, hypoxia, and more severe inflammation, that suggests a role for the body’s inflammatory and hypoxia in the mechanisms accounting for cardiovascular complications in patients with CAP.

According to new clinical classification of myocardial infarction [23], most AMI during CAP hospitalization in geriatric patients may be classified as type 2. This type AMI secondary to ischaemia due to either increased oxygen demand or decreased supply. ﻿The actual mechanisms by which pneumonia triggers myocardial ischaemia ﻿have not yet been fully evaluated. Acute pneumonia can induce inflammatory changes in atherosclerotic plaques, demand ischemia, endothelial dysfunction, and procoagulant changes in blood [4, 6]. Accumulating evidence on the mechanisms of ACS after acute infections suggests that inflammatory activity is thought to play a key role in the pathogenesis of coronary events. Acute infections induce substantial inflammatory reactions, they can potentially contribute to the development of ACS [24–26]. Pneumonia contributes to the acute worsening of pre-existing cardiac conditions and can trigger new cardiac events. In pneumonia, a generalized inflammatory response is usually fully activated by the time patients present to hospital [27]. Previous studies suggests that systemic inflammatory response may cause inflammation in coronary arteries and their pre-existing atherosclerotic lesions [28]. Infections can induce pro inflammatory changes in the cellular composition of the atherosclerotic lesions and make them more vulnerable to cause coronary [28, 29]. Acute infections can also cause acute dysfunction and/or physical disruption of the endothelium [30, 31].

The study has some limitations. The first is the retrospective design of the study which resulted in some variables cannot be extracted from the electronic medical records. Data of peripheral arteriosclerosis evaluation were incomplete in respiratory ward and some patients in respiratory wards did not have BNP results at the time of admission, these were not available for our analyses. Since the D-Dimer detection was done in the respiratory ward by a analyzer used only in the respiratory department, the data was not recorded in the electronic medical records management system of our hospital. Meanwhile, partly of patients didn’t have a CURB-65 or PSI score, which are most commonly ﻿scales for assessment the severity of CAP, ﻿nevertheless, the variables in both scales were included in the study. Secondly, in our real-world study, treatment regimens were relatively individual, and few patients underwent coronary angiography which is why it was not easy to analyze the relationship between specific treatment regimens and clinical outcomes. Future research will be required to determine if specific measures such as Aspirin or drugs lowering systemic inflammation could prevented cardiovascular complications in CAP inpatients. Thirdly, this study was conducted in a single hospital serving an urban area. It would be interesting to extend these observations in a larger sample and multicenter.

## Conclusions

The incidence of AMI during CAP hospitalization in geriatric patients is notable and have an impact on in-hospital mortality. Particular attention should be paid to older patients with respiratory failure, preexisting coronary artery disease, diabetes, with high BUN level and impaired consciousness. Our study may represent useful information for planning of clinical strategies aimed at preventing AMI and decreasing mortality rate in geriatric patients hospitalization for CAP.

## Data Availability

The datasets of the current study are available from the corresponding author on reasonable request.

## Abbreviations

CAP: Community-acquired pneumonia
AMI: Myocardial infarction cardiovascular
BUN: Blood urea nitrogen
OR: The odds ratios
CI: Confidence intervals
ACS: Acute coronary syndromes
IDSA/ATS: Infectious Diseases Society of America and the American Thoracic Society
PO_2_: Pressure of oxygen
PCO_2_: Pressure of carbon dioxide
IMV: Invasive mechanical ventilation
NIV: Non-invasive ventilation
PLT: Blood platelet
PH: Potential of hydrogen
